# Supplementary material for: Replication-IDentifier links epigenetic and metabolic pathways to the replication stress response
Source: Nat Commun. 2025 Feb 6;16:1416. doi: 10.1038/s41467-025-56561-y (PMC11802883; doi:10.1038/s41467-025-56561-y)
Supplement: Supplementary file 1 — Supplementary Information [file 41467_2025_56561_MOESM1_ESM.pdf]

Replication-IDentifier links epigenetic and metabolic pathways to the replication stress response

Sophie C. van der Horst, Leonie Kollenstart, Amandine Batté, Sander Keizer, Kees Vreeken, Praveen Pandey, Andrei Chabes and Haico van Attikum

This file includes:

- Supplementary Fig. 1
- Supplementary Fig. 2
- Supplementary Fig. 3
- Supplementary Fig. 4
- Supplementary Fig. 5
- Supplementary Fig. 6
- Supplementary Table 1
- Supplementary Table 2
- References

Van der Horst_Kollenstart et al_Supplementary Fig. 1

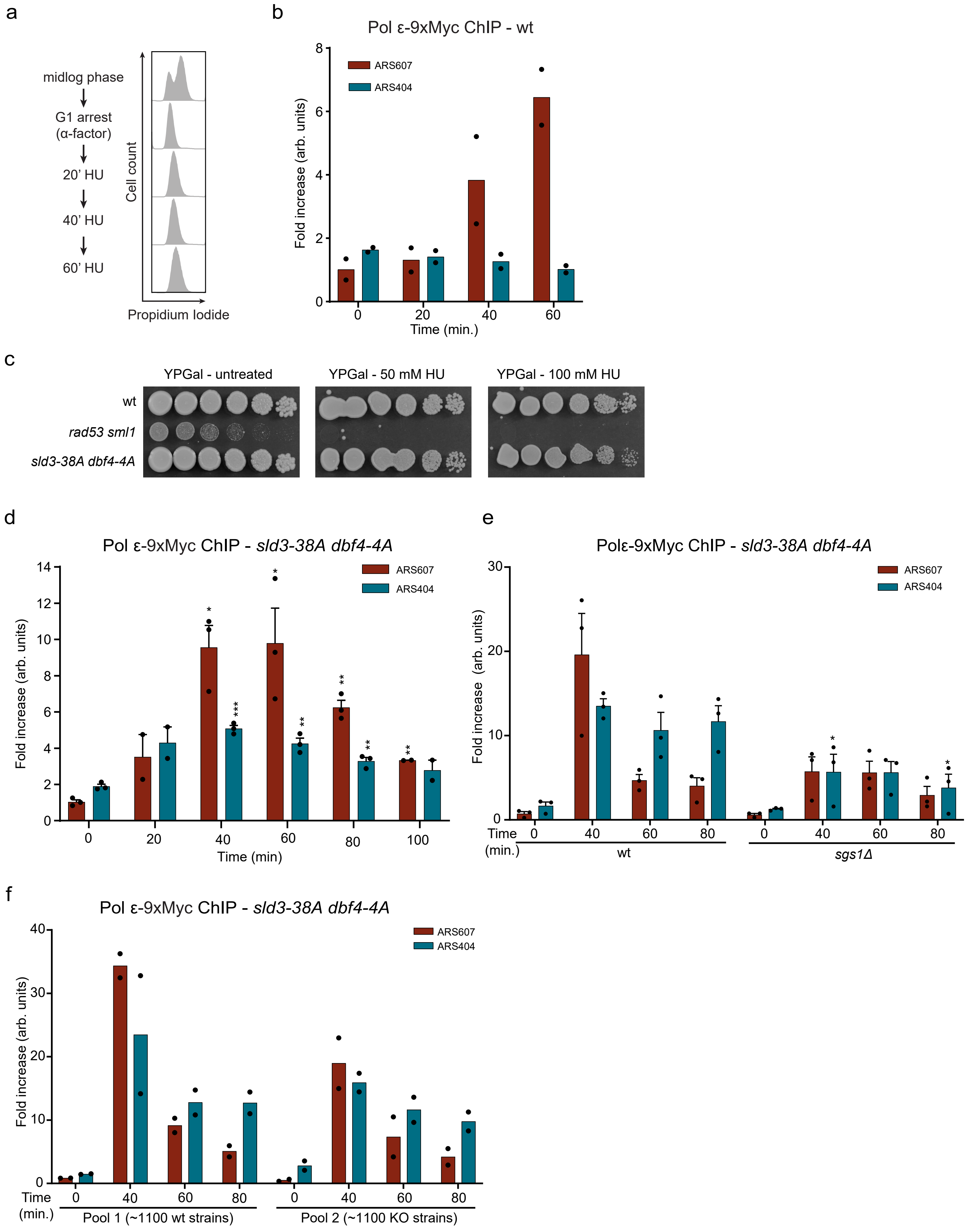

Supplementary Fig. 1: Optimization of Repli-ID conditions.

a Set-up of the ChIP experiments to study protein binding at ARS607 and ARS404. Left panel depicts the synchronized release of cells from G1 arrest into S-phase in the presence of HU. **b** ChIP-qPCR analysis of Pol ε-9xMyc at ARS607 and ARS404 in wild-type cells at different timepoints after G1 arrest and release in S-phase in YPAD + 200 mM HU. Data represent the mean relative fold enrichment + SEM of antibody signal over IgG signal in n=2 independent experiments. Values were normalized to a non-replicated region (ARS607+14kb). **c** Spot dilution assay with the indicated strains of the BY4741 background. Ten-fold serial dilutions were spotted onto rich YPAD medium containing galactose, with and without the indicated concentrations of HU. **d** As in **b**, except in *sld4-38A dbf4-4A* cells and in YPA + raffinose/galactose + 200 mM HU (n=2 or n=3). Statistical significance compared to t = 0 minutes was calculated using the two-tailed unpaired Student's t test, assuming unequal variances, $^{*}p < 0.05$, $^{**}p < 0.01$, $^{***}p < 0.001$. **e** As in b, except in *sld4-38A dbf4-4A* and *sgs1Δ sld4-38A dbf4-4A* cells and in YPA + raffinose/galactose + 200 mM HU (n=3). Statistical significance compared to *sld4-38A dbf4-4A* was calculated using the two-tailed unpaired Student's t test, assuming unequal variances, $^{*}p < 0.05$, $^{**}p < 0.01$, $^{***}p < 0.001$. **f** As in **b**, except in pooled barcoded *sld4-38A dbf4-4A* (wt) cells and *sld4-38A dbf4-4A* cells carrying different gene deletions (mutants) and in YPA + raffinose/galactose + 200 mM HU (n=2). Source data are provided as a Source Data file.

Van der Horst_Kollenstart et al_Supplementary Fig. 2

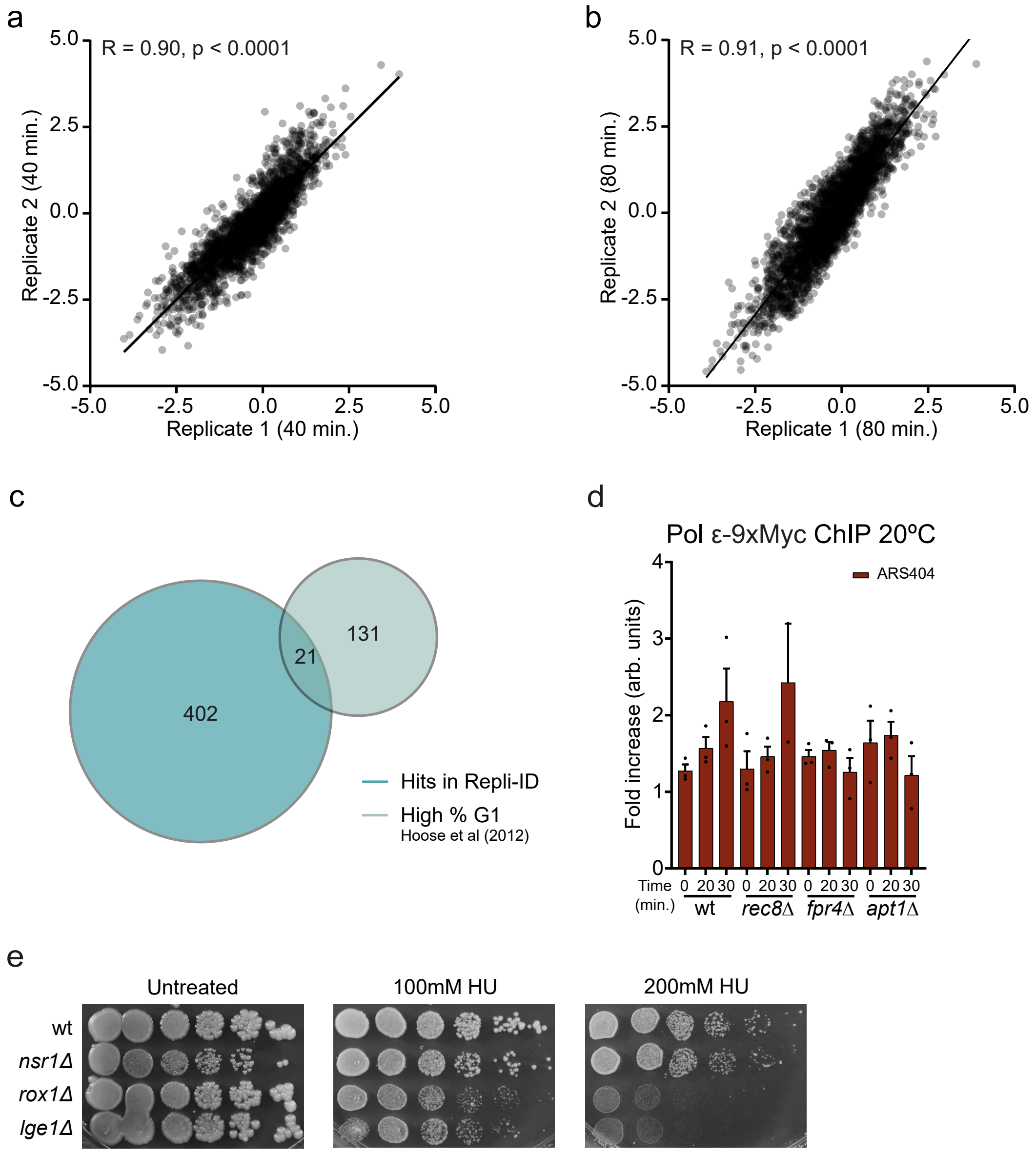

Supplementary Fig. 2: Quality controls and validation of Repli-ID screens.

a Scatter plot of the n=2 individual replicates of the Repli-ID screen for t=40 minutes. Spearman product moment correlation coefficient (R) with p-value is indicated. **b** As in **a**, except for t=80 minutes. **c** Venn Diagram[1] comparing hits from the Repli-ID screen with a previously published list of mutants with increased levels of G1 cells Hoose et al. (2012)[2]. **d** ChIP-qPCR analysis of Pol ε-9xMyc at ARS404 in *sld4-38A dbf4-4A* (wt) cells and *sld4-38A dbf4-4A* cells carrying different gene deletions (*rec8Δ, fpr4Δ, apt1Δ*) at different timepoints after G1 arrest and release in S-phase at 20 degrees without HU. Data represent the mean relative fold enrichment + SEM of antibody signal over beads only signal in two or three independent experiments. Values were normalized to a non-replicated region (ARS607+14kb). **e** Spot dilution assay with the indicated strains which were generated *de novo* in the W303 background. Five-fold serial dilutions were spotted on medium without or with the indicated concentrations of HU. Source data are provided as a Source Data file.

Van der Horst_Kollenstart et al_Supplementary Fig. 3

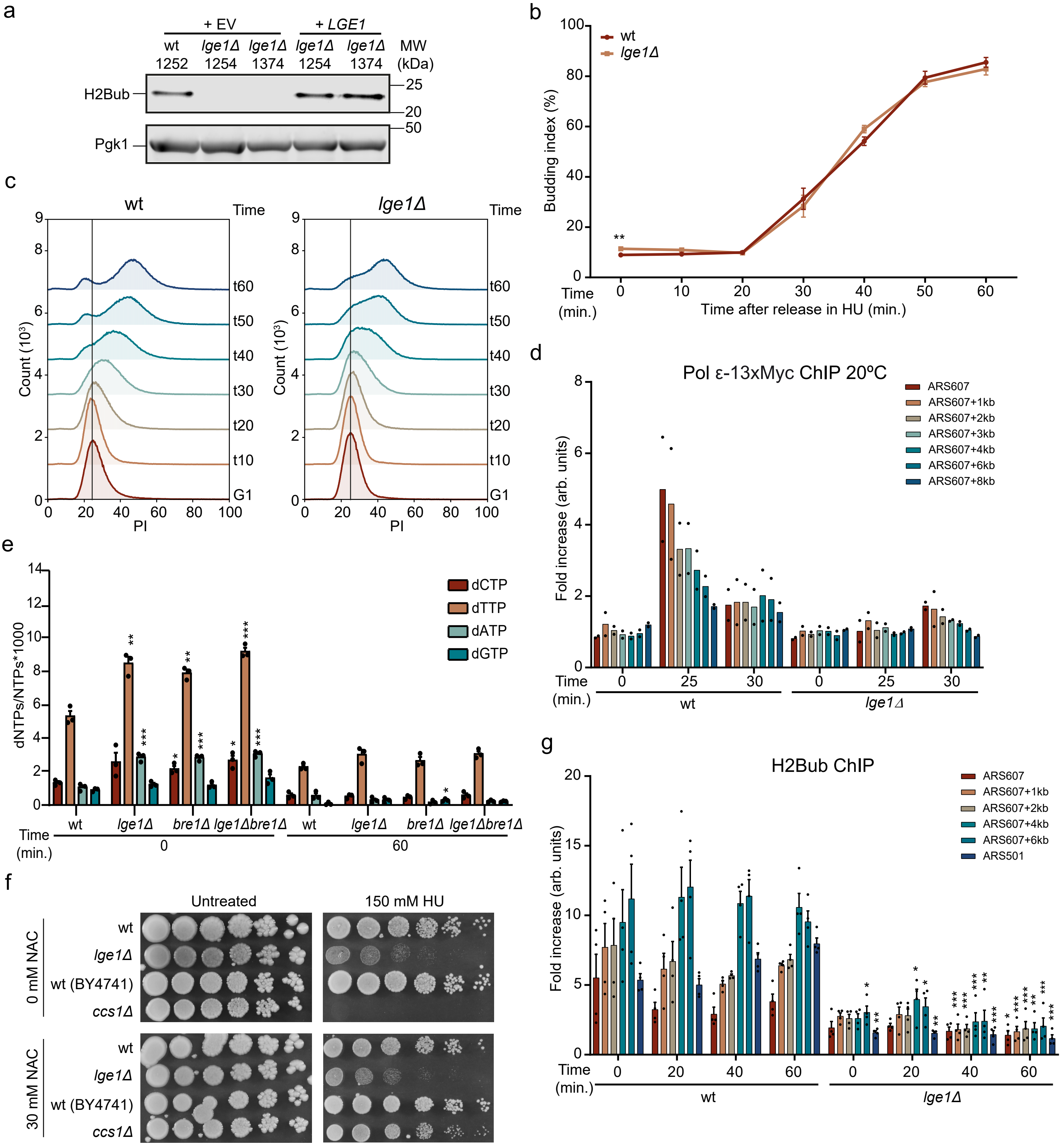

Supplementary Fig. 3: Impact of Lge1 loss on H2B ubiquitylation, Pol ε levels at ARS607 in unperturbed conditions, budding index, DNA copy number, dNTP levels, HU sensitivity in presence of NAC, and H2B ubiquitylation levels at ARS607 in presence of HU.

a Representative western blot analysis (n=2) of H2B ubiquitylation at Lysine 123 (H2Bub) in wt and two *lge1Δ* strains (yHA-1254 and yHA-1374) complemented by ectopic *LGE1* expression. Pgk1 is a loading control. **b** Budding index analysis of the indicated strains after G1 arrest and release in S-phase in 200 mM HU. Data represent the mean + SEM of three independent experiments. **c** Cell cycle profiling of the indicated strains. Cells were grown, arrested in G1 and released in 10mM of HU. DNA content was determined by propidium iodide (PI) staining and flow cytometry. Replicate of experiment shown in Fig. 3d. **d** ChIP-qPCR analysis of Pol ε-13xMyc near ARS607 in the indicated strains at different timepoints after G1-arrest and release S-phase at 20 degrees without HU. Data represent the mean relative fold enrichment of antibody signal over beads only signal in n=2 independent experiments. Values were normalized to a non-replicated region (ARS607+14kb). **e** dNTP analysis in the indicated strains after G1 arrest and a 60 minutes release in S-phase in 200 mM HU. Data represent the mean + SEM of three independent experiments. **f** Spot dilution assay with the indicated strains. Five-fold serial dilutions were spotted on medium without or with the indicated concentrations of HU and N-acetyl cysteine (NAC). *ccs1Δ* (with its parental strain BY4741) serves as a positive control **g** ChIP-qPCR analysis of H2B ubiquitylation at Lysine 123 (H2Bub) at ARS607 in the indicated strains at different timepoints after release from G1 in S-phase in 200 mM HU. Data represent the mean relative fold enrichment + SEM of H2Bub signal over input signal of n=3 independent experiments. Values were normalized to a telomere region (TELVI-R) Statistical significance compared to wt was calculated using the two-tailed unpaired Student's t test, assuming unequal variances, $*p < 0.05$, $**p < 0.01$, $***p < 0.001$. Source data are provided as a Source Data file.

Van der Horst_Kollenstart et al_Supplementary Fig. 4

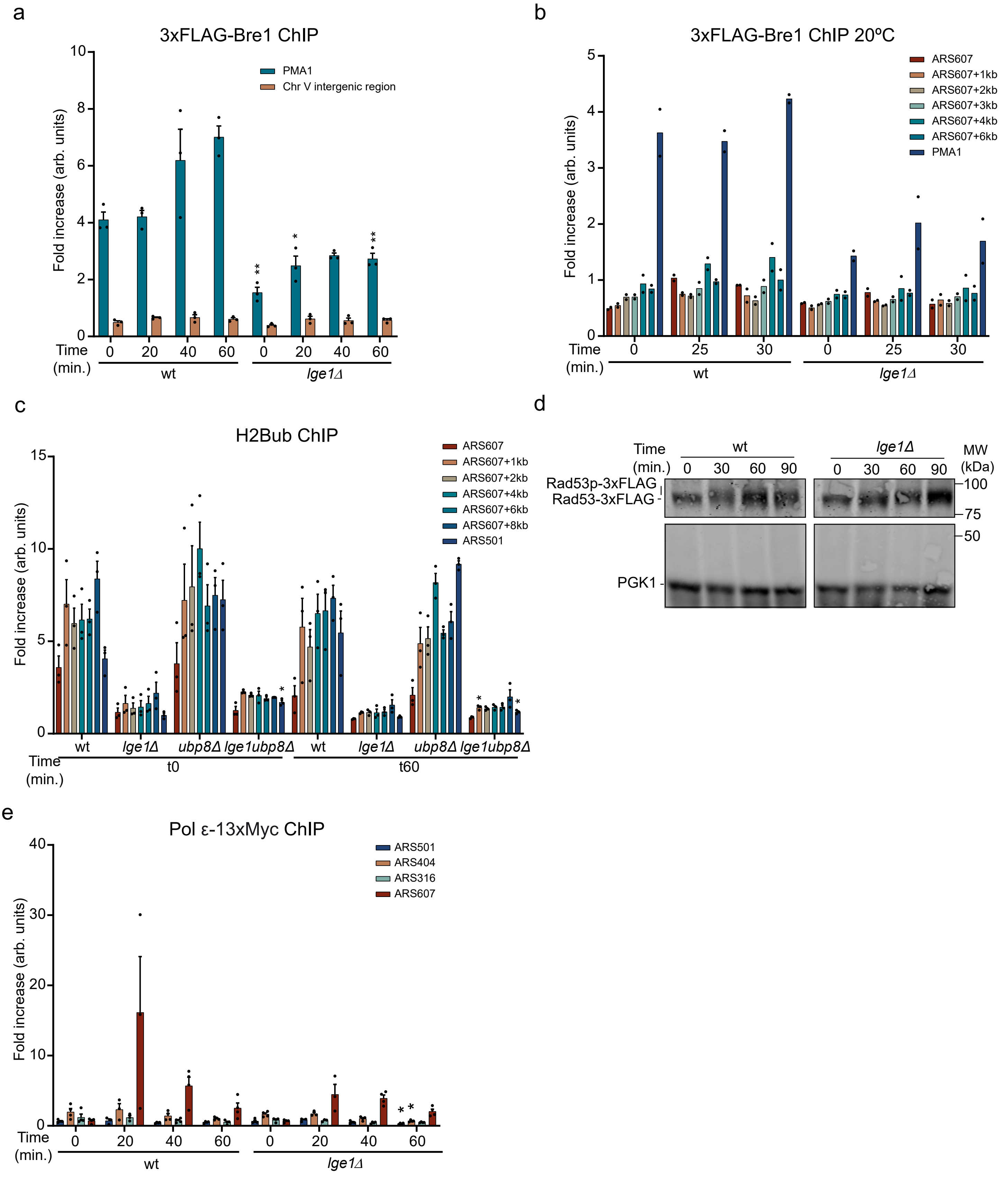

Supplementary Fig. 4: Lge1 loss impacts Bre1 loading at ARS607, but not at a transcription site, affects H2B ubiquitylation levels at ARS606 independently of Upb8, and does not lead to premature firing of late origins.

a ChIP-qPCR analysis of 3xFLAG-Bre1 at the *PMA1* loucs and Chr V intergenic region in the indicated strains at different timepoints after release from G1 in S-phase in 200 mM HU. Data represent the mean relative fold enrichment + SEM of FLAG signal over beads only signal of n=3 independent experiments. Values were normalized to a telomere region (TELVI-R). **b** As in **a**, except at ARS607 and PMA1 at different timepoints after release from G1 in S-phase at 20ºC without HU. Data represent the mean relative fold enrichment + SEM of FLAG signal over beads only signal of n=2 independent experiments. **c** as in **a,** except for H2B ubiquitylation at Lysine 123 (H2Bub) at ARS607 and ARS501. Data represent the mean relative fold enrichment + SEM of H2Bub signal over input signal of n=3 independent experiments. **d** Western blot analysis (n=2) of Rad53-3xFLAG phosphorylation in the indicated strains at different timepoints after G1 arrest and release in S-phase in 200 mM HU. Pgk1 is a loading control. Replicate of experiment shown in Fig. 4b. **e** as in **a,** except for Pol ε-13xMyc at ARS501, ARS404, ARS316 and ARS607. Data represent the mean relative fold enrichment + SEM of Myc signal over beads only signal of n=3 or n=4 independent experiments. Values were normalized to a non-replicated region (ARS607+14kb). Statistical significance compared to wt was calculated using the two-tailed unpaired Student's t test, assuming unequal variances, $*p < 0.05$, $**p < 0.01$, $***p < 0.001$. Source data are provided as a Source Data file.

Van der Horst_Kollenstart et al_Supplementary Fig. 5

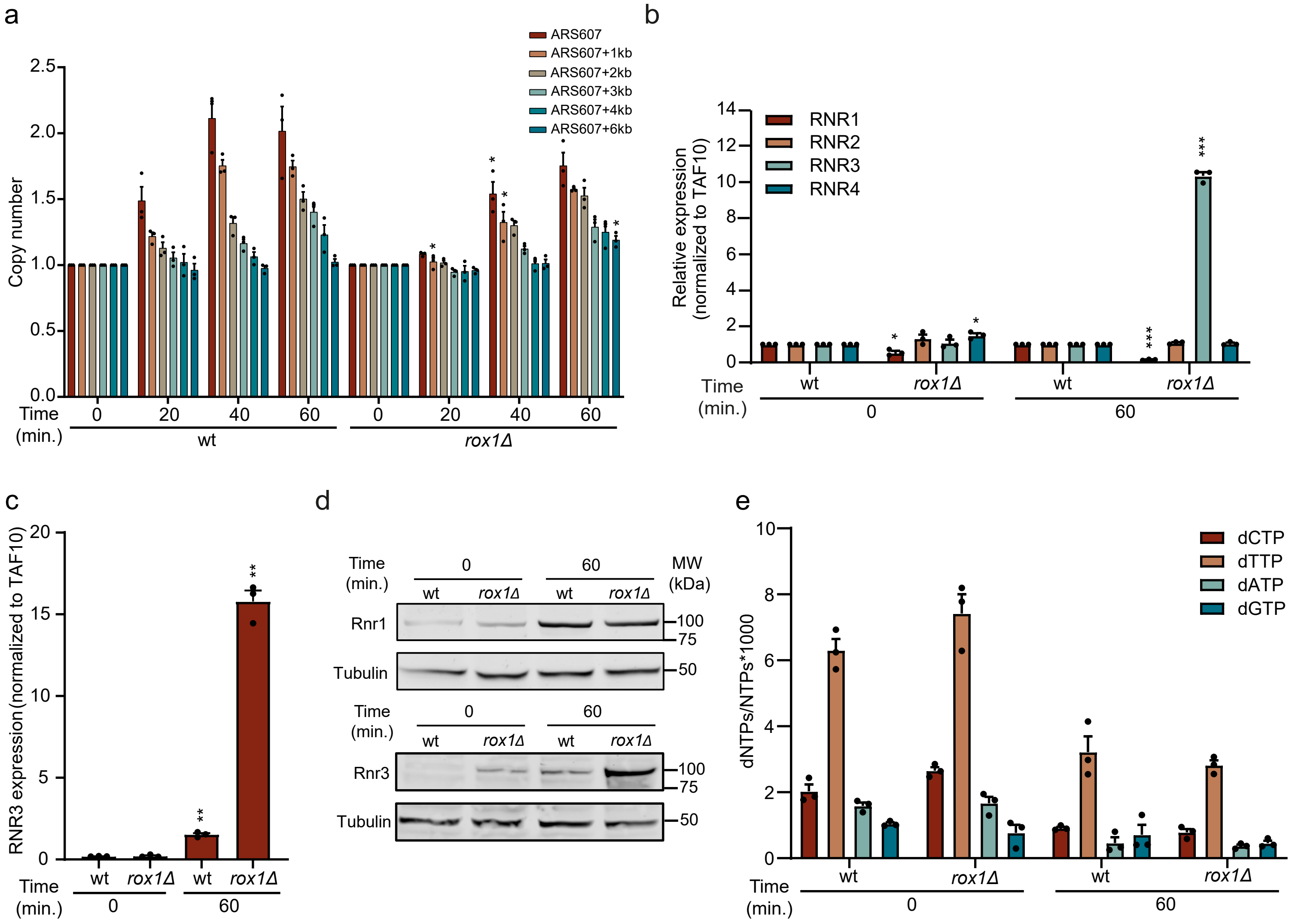

Supplementary Fig. 5: Rox1 does not affect DNA synthesis by regulating dNTP levels. **a** Dynamics of ARS607 duplication assayed by DNA copy number analysis using qPCR in the indicated strains at different timepoints after release from G1 in S-phase in 200 mM HU. Data represent the mean DNA quantity + SEM of n=3 independent experiments. Values were normalized to a non-replicated region (ARS607+14kb) and further normalized to the ratio of the samples in G1, which were set to 1. **b** RT-qPCR analysis of *RNR1*, *RNR2*, *RNR3* and *RNR4* expression in the indicated strains after G1 arrest and a 60 minutes release in S-phase in 200 mM HU. Data represent the mean expression relative to that of *TAF10,* which served as a reference, and to that in wt, which was set to 1, + SEM in n=3 independent experiments. **c** RT-qPCR analysis of *RNR3* expression in the indicated strains after G1 arrest and a 60 minutes release in S-phase in 200 mM HU. Data represent the mean expression relative to that of TAF10, which served as a reference, + SEM in n=3 independent experiments. **d** Representative western blot analysis (n=2) of Rnr1 and Rnr3 expression in the indicated strains after G1 arrest and a 60 minutes release in S-phase in 200 mM HU. Tubulin is a loading control. **e** dNTP analysis in the indicated strains after G1 arrest and a 60 minutes release in 200 mM HU. Data represent the mean + SEM of n=3 independent experiments. Statistical significance compared to wt was calculated using the two-tailed unpaired Student's t test, assuming unequal variances, $*p < 0.05$, $**p < 0.01$, $***p < 0.001$. Source data are provided as a Source Data file.

Van der Horst_Kollenstart et al_Supplementary Fig. 6

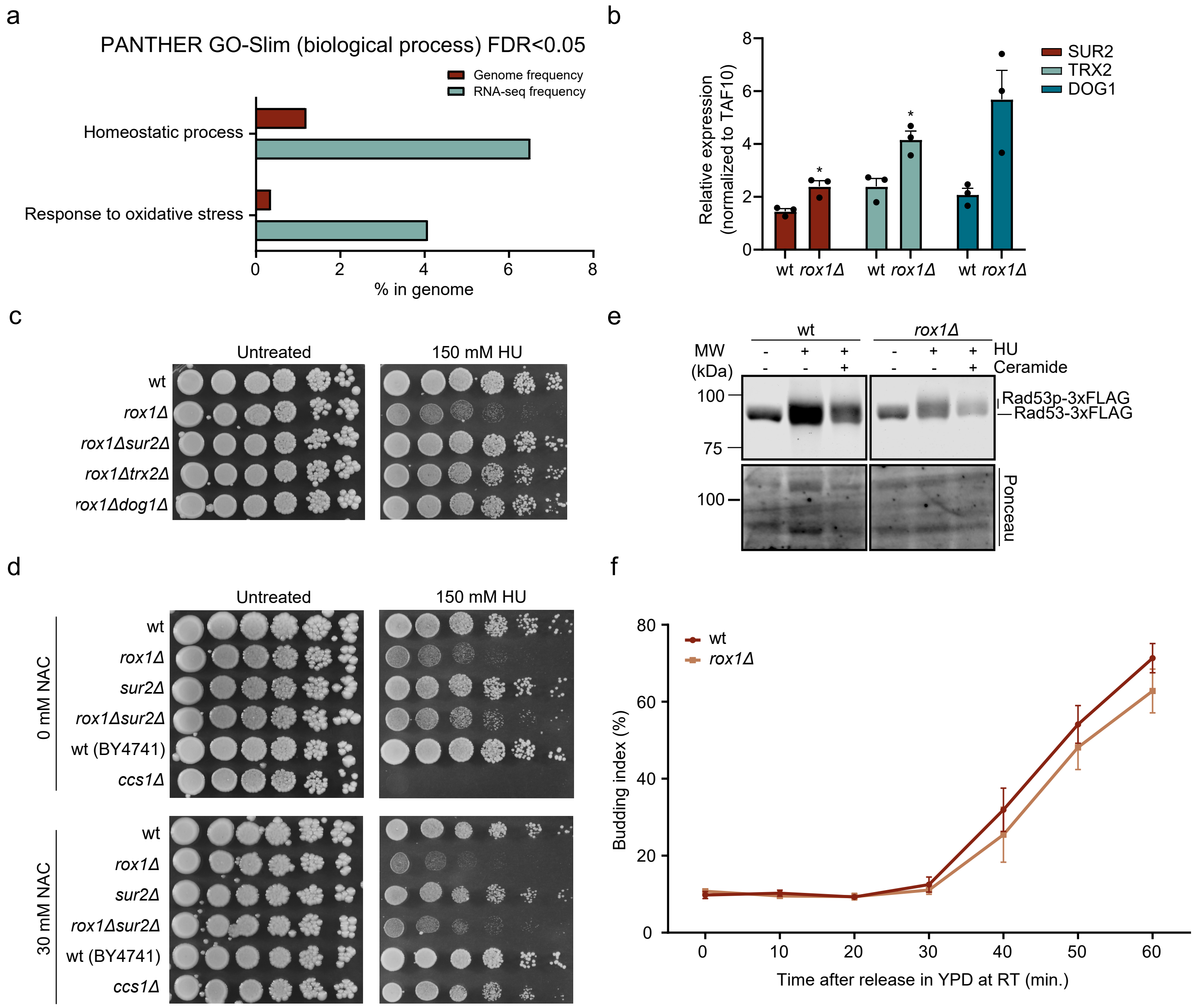

Supplementary Fig. 6: Validation of RNA-seq and suppressor screens, NAC does not impact the HU sensitivity of *rox1Δ,* and S-phase entry is unaffected in *rox1Δ* in unperturbed conditions.

a Panther GO Slim biological process analysis (FDR<0.05) of the top 137 genes showing higher expression in *rox1Δ* (Fold change > 1.5 and $p < 0.05$). Genome frequency is depicted in red and frequency in the RNA-seq data in blue. **b** Validation of RNA-seq by RT-qPCR analysis of *SUR2*, *TRX2* and *DOG1* expression in the indicated strains after G1 arrest and a 60 minutes release in S-phase in 200 mM HU. Data represent the mean expression relative to that of *TAF10* expression + SEM in n=3 independent experiments. **c** Spot dilution assay with the indicated mutants from the suppressor screen. Five-fold serial dilutions were spotted on medium without or with the indicated concentration of HU. **d** Spot dilution assay with the indicated strains. Five-fold serial dilutions were spotted on medium without or with the indicated concentration of HU and N-acetyl cysteine (NAC). *ccs1Δ* (with its parental strain BY4741) served as a positive control. **e** Western blot analysis (n=2) of Rad53-3xFLAG phosphorylation in the indicated strains after G1 arrest and a 60 minutes release in S-phase in 200 mM HU and/or 15 µM ceramide. Total protein staining by Ponceau is a loading control. Replicate of experiment shown in Fig. 5b. **f** Budding index analysis of the indicated strains after G1 arrest and release in S-phase at room temperature (RT). Data represent the mean + SEM of n=3 independent experiments. Statistical significance compared to wt was calculated using the two-tailed unpaired Student's t test, assuming unequal variances, $^{*}p < 0.05$, $^{**}p < 0.01$, $^{***}p < 0.001$. Source data are provided as a Source Data file.

Supplementary Table 1: Yeast strains

Name	Genotype	Reference
yHA-1090	*MATα his3-1 leu2-0 lys2+ met15-0 ura3-0 Pol2-9xMyc-HPH*	This study
yHA-1115	*MATα his3-1 leu2-0 lys2+ met15-0 ura3-0 can1::STE2pr-SpHIS5, lyp1::STE3pr-LEU2, cyh2, Pol2-9xMyc-HPH, bar1::Dbf4-4A-pGAL1-10-Sld3-38A-URA3*	This study
yHA-269	*MATa ade2-1 trp1-1 his3-11 his3-15 ura3-1 leu2-3 leu2-112 rad5-G535R Pol2-13xMyc-KanMX bar1Δ::NatMX* (W303)	[3]
yHA-1167	yHA-269, except *nsr1Δ::HIS3*	This study
yHA-1168	yHA-269, except *fpr4Δ::HIS3*	This study
yHA-1169	yHA-269, except *tpa1Δ::HIS3*	This study
yHA-1170	yHA-269, except *pml1Δ::HIS3*	This study
yHA-1171	yHA-269, except *rox1Δ::HIS3*	This study
yHA-265	*MATa ade2-1 trp1-1 his3-11 his3-15 ura3-1 leu2-3 leu2-112 bar1Δ::LEU2 DDC2-YFP* (W303)	[4]
yHA-1243	yHA-265, except *rox1Δ::HIS3*	This study
yHA-1423	yHA-265, except *sur2Δ::URA3*	This study
yHA-1424	yHA-265, except *rox1Δ::HIS3 sur2Δ::URA3*	This study
yHA-1425	yHA-265, except *trx2Δ::URA3*	This study
yHA-1426	yHA-265, except *rox1Δ::HIS3 trx2Δ::URA3*	This study
yHA-1427	yHA-265, except *dog1Δ::URA3*	This study
yHA-1428	yHA-265 except *rox1Δ::HIS3 dog1Δ::URA3*	This study
yHA-1164	yHA-265 except *Mcm4-3xFLAG::HPH*	This study
yHA-1455	yHA-265 except *Mcm4-3xFLAG::HPH rox1Δ::HIS*	This study
yHA-1456	yHA-265 except *Mcm4-3xFLAG::HPH lge1Δ::HIS*	This study
yHA-1457	yHA-265, except *rox1Δ::HIS3 irc21Δ::HPH*	This study
yHA-1458	yHA-265, except *rox1Δ::HIS3 irc21Δ::HPH*	This study
yHA-1459	yHA-265, except *rox1Δ::HIS3 sur2Δ::URA3 irc21Δ::HPH*	This study
yHA-1249	*MATa ade2-1 trp1-1 his3-11 his3-15 ura3-1 leu2-3 leu2-112 Pol2-13xMyc-KanMX bar1Δ::NatMX RAD53-3xFLAG-HPH* (W303)	This study
yHA-1254	yHA-1249, except *lge1Δ::HIS3*	This study
yHA-1395	yHA-1249, except *bre1Δ::HIS3*	This study
yHA-1431	yHA-1249, except *rox1Δ::HIS3*	This study
yHA-1432	yHA-1249, except *sur2Δ::URA3*	This study
yHA-1433	yHA-1249, except *rox1Δ::HIS3 sur2Δ::URA3*	This study
yHA-1252	*MATa ade2-1 trp1-1 his3-11 his3-15 ura3-1 leu2-3 leu2-112 bar1Δ::NatMX* (W303)	This study
yHA-1372	yHA-1252 except *bre1Δ::HIS3*	This study
yHA-1374	yHA-1252 except *lge1Δ::HIS3*	This study
yHA-1376	yHA-1252 except *bre1Δ::HIS3 lge1Δ::URA3*	This study
yHA-1397	yHA-1252 except *bre1Δ::HIS3 3xFLAG-BRE1-LEU2::leu2-3 leu2-112*	This study
yHA-1398	yHA-1252 except *bre1Δ::HIS3 lge1Δ::URA3 3xFLAG-BRE1-LEU::leu2-3 leu2-112*	This study
yHA-1452	yHA-1252 except *ubp8Δ::HPH*	This study
yHA-1454	yHA-1252 except *lge1Δ::HIS3 ubp8Δ::HPH*	This study

Supplementary Table 2: qPCR Primers

Target	Sequence
ARS607 fw	CTTTAGCTGGGTTTATGGGAGGTT
ARS607 rv	TAATGCACGAGCCGAAACAA
ARS607+1kb fw	GGAGAGAATCTTACCTCAGAGTGC
ARS607+1kb rv	GGGATCTTGAAAGTAAAACAGGTG
ARS607+2kb fw	CGCAGCAGTGGAGTTATCAG
ARS607+2kb rv	TAATCCACTTTGTCGGGCCA
ARS607+3kb fw	CTTTGTTATGGACCCGGAGA
ARS607+3kb rv	CATCAAGATGGAATACTGTGACAA
ARS607+4kb fw	TATGCTATCGTCGAGATGTTGTTCT
ARS607+4kb rv	GGTGGAAGCGCAGGTTGATC
ARS607+6kb fw	GTTTCACCTCGTAGTCCCTCA
ARS607+6kb rv	AACCAAATGCATTGCTTTATCA
ARS607+8kb fw	AAGTTGGGTCAAAGAGAAAGG
ARS607+8kb rv	CTCTCCATTGCTTCCCTAAAAC
ARS607+14kb fw	CAGGATATGCGGCCAAATTT
ARS607+14kb rv	GCATGACAGCCGAATCGAT
ARS501 fw	AAGCAAATTGCAGAAGGTTATGAA
ARS501 rv	TTCAAGGCTCTAGCATATGAAACG
ARS305 fw	CGCCCGACGCCGTAA
ARS305 rv	GAGCGGCCTGAAATACTGTCA
ARS305+2.6kb fw	CAAAGGTCGGCTGCTTCAAT
ARS305+2.6kb rv	GGTATAGGCCAGGGAAGAAGGT
ARS404 fw (HOtermQfw[5])	GAGTAGAAATACGCCATCTCAAGATACA
ARS404 rv (HOtermQrv[5])	GGAAAGTTGATCAAGACCCAATAATAA
TELVI-R fw	GGCTGGACTACTTTCTGGAATAGC
TELVI-R rv	GAACTGTGCATCCACTCGTTAGG
RNR1 fw	ATGCCTACTGCATCCACATC
RNR1 rv	GACACGACGGGAGTACATATTG
RNR2 fw	CTTTGCCTCCATTGAAGGTG
RNR2 rv	GTTAAACCGGGCATCATACC
RNR3 fw	GCCTCCGCTGCTATTCAA
RNR3 rv	CAGATGCCGCCTTTTGTT
RNR4 fw	GCATTTGCTGCAAAGGAAGG
RNR4 rv	GTTGGCCATTGCTAAACCTG
SUR2 fw	GCACTTCGAGCCGATCTAC
SUR2 rv	GCGGACATTCCGTACATATAGC
TRX2 fw	GCTGAAGTTTCTTCCATGCC
TRX2 rv	CGTTGGAAGCAATAGCTTGC
DOG1 fw	GTGAGTACAACAGTGGCCG
DOG1 rv	CTCAAAACCTCTTGTGTTCTTGC
TAF10 fw	ATATTCCAGGATCAGGTCTTCCGTAGC
TAF10 rv	GTAGTCTTCTCATTCTGTTGATGTTGTTGTTG
PMA1 fw (adapted from[6])	CGACGACGAAGACAGTGATAAC
PMA1 rv (adapted from[6])	CGTCAGCCATTTGATTCAAACC
Intergenic region Chr V (adapted from[6])	GTGAGATTCTTCCTGGCTGTC
Intergenic region Chr V (adapted from[6])	CAATACAGTGCGCAGTACTTG
ARS316 fw (from[7])	CGGCATTATCGTACACAACCT
ARS316 rv (from[7])	GTTCTTCGTTGCCTACATTTTCT

References

1. Hulsen, T., de Vlieg, J. & Alkema, W. BioVenn – a web application for the comparison and visualization of biological lists using area-proportional Venn diagrams. *BMC Genomics* **9**, 488 (2008).
2. Hoose, S.A. *et al.* A Systematic Analysis of Cell Cycle Regulators in Yeast Reveals That Most Factors Act Independently of Cell Size to Control Initiation of Division. *PLOS Genetics* **8**, e1002590 (2012).
3. Batté, A. *et al.* Chl1 helicase controls replication fork progression by regulating dNTP pools. *Life Sci Alliance* **5**, e202101153 (2022).
4. Lisby, M., Barlow, J.H., Burgess, R.C. & Rothstein, R. Choreography of the DNA damage response: spatiotemporal relationships among checkpoint and repair proteins. *Cell* **118**, 699-713 (2004).
5. Vlaming, H. *et al.* Direct screening for chromatin status on DNA barcodes in yeast delineates the regulome of H3K79 methylation by Dot1. *eLife* **5**, e18919 (2016).
6. Song, Y.H. & Ahn, S.H. A Bre1-associated protein, large 1 (Lge1), promotes H2B ubiquitylation during the early stages of transcription elongation. *J Biol Chem* **285**, 2361-7 (2010).
7. Weiβ, M. *et al.* Single-copy locus proteomics of early- and late-firing DNA replication origins identifies a role of Ask1/DASH complex in replication timing control. *Cell Rep* **42**, 112045 (2023).
