# Supplementary material for: Replication-IDentifier links epigenetic and metabolic pathways to the replication stress response
Source: Nat Commun. 2025 Feb 6;16:1416. doi: 10.1038/s41467-025-56561-y (PMC11802883; doi:10.1038/s41467-025-56561-y)
Supplement: Supplementary file 2 — Reporting Summary [file 41467_2025_56561_MOESM2_ESM.pdf]

nature portfolio

Corresponding author(s): Haico van Attikum

Last updated by author(s): 06/01/2025

Reporting Summary

Nature Portfolio wishes to improve the reproducibility of the work that we publish. This form provides structure for consistency and transparency in reporting. For further information on Nature Portfolio policies, see our Editorial Policies and the Editorial Policy Checklist.

Statistics

For all statistical analyses, confirm that the following items are present in the figure legend, table legend, main text, or Methods section.

n/a	Confirmed	
☐	☒	The exact sample size (n) for each experimental group/condition, given as a discrete number and unit of measurement
☐	☒	A statement on whether measurements were taken from distinct samples or whether the same sample was measured repeatedly
☐	☒	The statistical test(s) used AND whether they are one- or two-sided<br>*Only common tests should be described solely by name; describe more complex techniques in the Methods section.*
☒	☐	A description of all covariates tested
☒	☐	A description of any assumptions or corrections, such as tests of normality and adjustment for multiple comparisons
☐	☒	A full description of the statistical parameters including central tendency (e.g. means) or other basic estimates (e.g. regression coefficient) AND variation (e.g. standard deviation) or associated estimates of uncertainty (e.g. confidence intervals)
☐	☒	For null hypothesis testing, the test statistic (e.g. F, t, r) with confidence intervals, effect sizes, degrees of freedom and P value noted<br>*Give P values as exact values whenever suitable.*
☒	☐	For Bayesian analysis, information on the choice of priors and Markov chain Monte Carlo settings
☒	☐	For hierarchical and complex designs, identification of the appropriate level for tests and full reporting of outcomes
☒	☐	Estimates of effect sizes (e.g. Cohen's d, Pearson's r), indicating how they were calculated

Our web collection on statistics for biologists contains articles on many of the points above.

Software and code

Policy information about availability of computer code

Data collection	Licor Odyssey V3.0 (Western blots)<br>Singer Instruments ROTOR (Repli-ID screen and suppressor screen)<br>Biorad CFX384 Touch Real-Time PCR Detection System (qPCR)<br>Zeiss AxioImager M2 widefield fluorescence microscope (Budding index experiments)
Data analysis	RStudio 2023.09.1 (Repli-ID screen analysis)<br>xcalibr https://github.com/NKI-GCF/xcalibr as in Vlaming et al (2016) (Repli-ID screen analysis)<br>PANTHER GO enrichment analysis https://geneontology.org (GO term analysis)<br>BioRad CFX Manager Software 3.1 (qPCR)<br>LI-COR ImageStudio Lite 5.2.5 (western blot)<br>usegalaxy.org using TRIMMOMATIC, HISAT2, FEATURECOUNTS and DESEQ2 (RNA-seq analsyis)<br>SGAtools (http://sgatools.ccbr.utoronto.ca/) (Suppressor screen)<br>GraphPad Prism 6 (plotting and statistics)<br>SnapGene 7.1.1 (DNA cloning design and sequence alignment)<br>Image J 1.48v (Microscopy image analysis)<br>ZEN 2012 microscopy software (wide-field microscopy)

For manuscripts utilizing custom algorithms or software that are central to the research but not yet described in published literature, software must be made available to editors and reviewers. We strongly encourage code deposition in a community repository (e.g. GitHub). See the Nature Portfolio guidelines for submitting code & software for further information.

Data

Policy information about availability of data

All manuscripts must include a data availability statement. This statement should provide the following information, where applicable:

- Accession codes, unique identifiers, or web links for publicly available datasets
- A description of any restrictions on data availability
- For clinical datasets or third party data, please ensure that the statement adheres to our policy

The Repli-ID screen data generated in this study have been deposited in the Sequence Read Archive of the National Center for Biotechnology Information database under accession code SRR28063796 (PRJNA1079539): https://www.ncbi.nlm.nih.gov/bioproject/PRJNA1079539.

TThe RNA-seq data generated in this study have been deposited in the Sequence Read Archive of the National Center for Biotechnology Information database under accession codes SRR27973084 - SRR27973094 (PRJNA1076696) : https://www.ncbi.nlm.nih.gov/bioproject/PRJNA1076696.

All other data generated in this study are provided in the Supplementary Information/Source Data file. Source data are provided with this paper.

Research involving human participants, their data, or biological material

Policy information about studies with human participants or human data. See also policy information about sex, gender (identity/presentation), and sexual orientation and race, ethnicity and racism.

Reporting on sex and gender	n/a
Reporting on race, ethnicity, or other socially relevant groupings	n/a
Population characteristics	n/a
Recruitment	n/a
Ethics oversight	n/a

Note that full information on the approval of the study protocol must also be provided in the manuscript.

Field-specific reporting

Please select the one below that is the best fit for your research. If you are not sure, read the appropriate sections before making your selection.

[x] Life sciences [ ] Behavioural & social sciences [ ] Ecological, evolutionary & environmental sciences

For a reference copy of the document with all sections, see nature.com/documents/nr-reporting-summary-flat.pdf

Life sciences study design

All studies must disclose on these points even when the disclosure is negative.

Sample size	Sample sizes were determined based on previous experience and common practice in the field. For experiments in which qPCR was used (ChIP-qPCR, copy number analysis, RT-qPCR), enough cells were seeded to retrieve Ct values between 15-30/35. For budding index experiments, at least 200 cells were analyzed per sample in order to obtain reliable and reproducible results. Viability tests were performed with at least 50 colonies per plate at timepoint t0 and three technical replicates were counted per experiment for each condition. For FACS experiments 250.000 events were recorded.
Data exclusions	No data were excluded.
Replication	Experiments were performed at least in duplicate, except for the suppressor screen (n=1), but mostly in triplicate or more to asses the reproducibility. All attempts at reproduction were successful. Standard errors included in the graphs indicate the variation between replicates of each experiment.
Randomization	Different samples from one experiment were treated as one group, so random allocation was not necessary in this study.
Blinding	Blinding has not been applied, because it is not feasible for many of the approaches used in our manuscript, including IPs and western blot analysis. Furthermore, when using qPCR and FACS, unbiased quantification was guaranteed by software-based analysis using identical parameters between conditions and/or normalization methods.

Reporting for specific materials, systems and methods

We require information from authors about some types of materials, experimental systems and methods used in many studies. Here, indicate whether each material, system or method listed is relevant to your study. If you are not sure if a list item applies to your research, read the appropriate section before selecting a response.

Materials & experimental systems

n/a	Involved in the study
☐	☒ Antibodies
☒	☐ Eukaryotic cell lines
☒	☐ Palaeontology and archaeology
☒	☐ Animals and other organisms
☒	☐ Clinical data
☒	☐ Dual use research of concern
☒	☐ Plants

Methods

n/a	Involved in the study
☒	☐ ChIP-seq
☒	☐ Flow cytometry
☒	☐ MRI-based neuroimaging

Antibodies

Antibodies used

Antibodies used were anti-Myc 9B11 (#2276; Cell Signaling; ChIP: 2µl per 40µl beads), anti-FLAG (F1804; Sigma; WB: 1:5000; ChIP: 2µl per 40µl beads), anti-Pgk1 (#459250; Invitrogen; WB: 1:5000), anti-H2Bub (received from Fred van Leeuwen, NKI, Amsterdam, described in van Welsem, et al. (2018); WB: 1:5000; ChIP: 0.2µl per 40µl beads), anti-RNR1 (AS214608; Agrisera; WB: 1:5000), anti-RNR3 (AS09574; Agrisera; WB: 1:1000), anti-tubulin (T6199; Sigma; WB: 1:1000).

Validation

Commercially available antibodies were validated by the supplier:
- anti-Pgk1 (#459250; Invitrogen), 467 citations on website of supplier
- anti-tubulin (T6199; Sigma), validated supplier's Antibody Enhanced validation method

The following antibodies were additionally validated by us (not shown in manuscript):
- anti-RNR1 (AS214608; Agrisera) and anti-RNR3 (AS09574; Agrisera) in wild-type versus RNR1 or RNR3 deletion mutant strains.
- anti-myc 9B11 (#2276; Cell Signaling), anti-FLAG (F1804; Sigma) in untagged wildtype versus Myc- or FLAG-tagged strains

anti-H2Bub was validated by van Welsum et al (2018) NAR.

Plants

Seed stocks

n/a

Novel plant genotypes

n/a

Authentication

n/a
