# Supplementary material for: Replication-IDentifier links epigenetic and metabolic pathways to the replication stress response
Source: Nat Commun. 2025 Feb 6;16:1416. doi: 10.1038/s41467-025-56561-y (PMC11802883; doi:10.1038/s41467-025-56561-y)
Supplement: Supplementary file 4 — Description of Additional Supplementary Files [file 41467_2025_56561_MOESM4_ESM.pdf]

File Name: Supplementary Data 1

Description: Outcome of Repli-ID for Pol ε-9xMyc in 2905 yeast mutants at t=40 and t=80 minutes. This table includes IP/input ratios of the mean of n=2 independent Repli-ID screens at 40 and 80 minutes after G1 arrest and release in 200 mM HU.
